# Setting of initiation and factors associated with antidepressant use on entry to long‐term care facilities

**DOI:** 10.1111/bcp.16403

**Published:** 2025-01-31

**Authors:** Georgina A. Hughes, Maria C. Inacio, Debra Rowett, Catherine Lang, Robert N. Jorissen, Megan Corlis, Janet K. Sluggett

**Affiliations:** ^1^ University of South Australia, UniSA Clinical & Health Sciences Adelaide South Australia Australia; ^2^ Registry of Senior Australians (ROSA), South Australian Health & Medical Research Institute Adelaide South Australia Australia; ^3^ University of South Australia, UniSA Allied Health & Human Performance Adelaide South Australia Australia; ^4^ Drug and Therapeutics Information Service, Southern Adelaide Local Health Network Adelaide South Australia Australia; ^5^ Australian Nursing & Midwifery Federation SA Branch Adelaide South Australia Australia

**Keywords:** aged, antidepressant, hospital, long‐term care, mirtazapine, nursing home

## Abstract

**Aims:**

Antidepressant use increases around long‐term care facility (LTCF) entry, and initiation during hospitalizations may contribute to this. This study characterized the care setting (i.e., community‐based, hospital or LTCF) where antidepressants were initiated and determined associated resident characteristics.

**Methods:**

A cross‐sectional study including non‐Indigenous individuals aged 65–105 years who entered LTCFs in two Australian states during 2015–2019, and were dispensed an antidepressant within 2 months, was conducted. Care settings (community‐based, hospital or LTCF) were determined from linked LTCF records, and hospitalizations ≤30 days before LTCF entry. Pharmaceutical claims before and after LTCF entry were screened to determine antidepressant initiation. Multivariate multinomial logistic regression estimated adjusted odds ratios (aORs) and 95% confidence intervals (95% CIs) for resident characteristics associated with care settings of antidepressant initiation.

**Results:**

This study included 34 525 residents from 1046 LTCFs. Overall, 27 160 (78.7%) commenced antidepressants prior to entry, 2552 (7.4%) in hospital and 4813 (13.9%) in LTCFs. Mirtazapine constituted 44.8% (*n* = 1143) of antidepressants initiated in hospitals and 39.5% (*n* = 1902) in LTCFs. Residents who were aged ≥90 years were more likely to start an antidepressant in the LTCF compared to community‐based settings (aOR = 1.97, 95% CI 1.74–2.23). Residents recently using a psychotropic were more likely to start an antidepressant in community‐based settings before LTCF entry, compared to a hospital or LTCF.

**Conclusions:**

Individuals receiving antidepressants during transition to LTCFs are often already taking antidepressants prior to entry. Future interventions to optimize antidepressant use in LTCFs should consider setting, recency and indication for antidepressant initiation, and ongoing monitoring for safety.

**What is already known about this subject**

- Approximately 60% of older people living in long‐term care facilities use an antidepressant.
- Antidepressant use increases around entry to long‐term care facilities.
- Transitions of care among older people can increase risk of harm, and untimely or incomplete handover between care providers can influence ongoing medicines management and continuity of care.

**What this study adds**

- Individuals receiving antidepressants during transition to long‐term care facilities often start the antidepressant before entry, including during hospitalization.
- Interventions to optimize antidepressant use in long‐term care facilities are needed, and should consider the setting and recency of antidepressant initiation, alongside indication for use and plans to monitor safety and effectiveness.

## 1 INTRODUCTION

Individuals living in long‐term care facilities (LTCFs; also known as nursing homes, residential aged care facilities or care homes) often experience multimorbidity, polypharmacy and frailty, and frequently transition between care settings.^1^ Older people entering LTCFs often transition directly from hospital, including more than half (57%) of new long‐term care residents in the United Kingdom, and 40% of assisted‐living residents in the United States (US) who require post‐acute care. In the US, 60% of post‐acute care is facility‐based and 10% transition to long‐term care within 6 months.^2^, ^3^, ^4^, ^5^ In Australia, pathways for entering long‐term care are often complex, with more than nine in ten people (94%) using home‐based, transition or respite aged care services before entering LTCFs permanently, along with 58% being hospitalized in the year before entry, and 10% in the fortnight prior.^6^, ^7^, ^8^ Transitions of care are particularly risky periods for medicines‐related harm.^9^ In accord with this, the World Health Organization (WHO) prioritized improvements to medicines safety among older people taking high‐risk medicines and during transitions of care, including movement between primary care, LTCFs and hospital settings.^10^

Recent hospitalization and entry to LTCFs are known risk factors for initiation of psychotropic medicines among older people.^11^, ^12^ Antipsychotic use in LTCFs, including initiation during hospitalization and interventions to reduce use, have received considerable focus globally^13^, ^14^, ^15^ and in Australia.^16^, ^17^ However, antidepressants have been examined to a lesser extent, and studies exploring antidepressant initiation around the time of LTCF entry have not investigated initiation among hospital inpatients prior to LTCF entry.^18^, ^19^, ^20^, ^21^, ^22^ An improved understanding of this area is necessary given six in ten Australian residents of LTCFs receive antidepressants annually, potentially exposing them to medicines‐related harms such as falls and fractures, urinary incontinence, cardiovascular events and other medicines‐related problems.^23^, ^24^, ^25^, ^26^, ^27^, ^28^ Hospitalizations can be distressing for older individuals and can have negative psychosocial impacts including loneliness, social isolation, depression and grief.^29^ Furthermore, health professionals may have minimal information on antidepressant indication and recency of initiation due to insufficient discharge documentation at transitions of care, which can further affect monitoring of antidepressant effectiveness and safety.^30^ The need for closer monitoring may not be readily identified post‐discharge as up to 76% of individuals entering LTCFs receive care from a new general medical practitioner (GP) and only 19% receive a comprehensive medicines review at LTCF entry.^31^, ^32^ Examining in‐hospital initiation of antidepressants is necessary to inform interventions to optimize the high and increasing use of antidepressants in LTCFs.^23^, ^33^

This study aimed to characterize the care setting (i.e., community‐based, hospital or LTCF) where antidepressants were commenced among older people who entered an LTCF and determine resident characteristics associated with initiation setting.

## 2 METHODS

### 2.1 Study design and data source

A cross‐sectional study was conducted using the Registry of Senior Australians (ROSA) National Historical Cohort. ROSA is a national integrated data platform with sociodemographic, medical, pharmaceutical, health and aged care service utilization and mortality information for individuals aged ≥65 years who have been assessed for eligibility and/or accessed government‐subsidized aged care services in Australia from 2002 onwards.^34^, ^35^ ROSA links information from national aged care datasets contained within the Australian Institute of Health and Welfare (AIHW) National Aged Care Data Clearinghouse (which includes the National Death Index), with the Australian Government Medicare Benefits Schedule (MBS, national government‐subsidized medical services), Pharmaceutical Benefits Scheme (PBS, government‐subsidized medicines) and hospital and emergency department claims data from selected Australian states.

This study analysed data from aged care eligibility and entry into LTCF assessments, episodes of aged care services, and medicines use from the PBS using PBS item codes and WHO Anatomical Therapeutic Chemical (ATC) classification codes.^36^ Hospital claims for two states (South Australia [SA], Victoria [VIC]) were analysed.

### 2.2 Study cohort and setting

**FIGURE 1:**
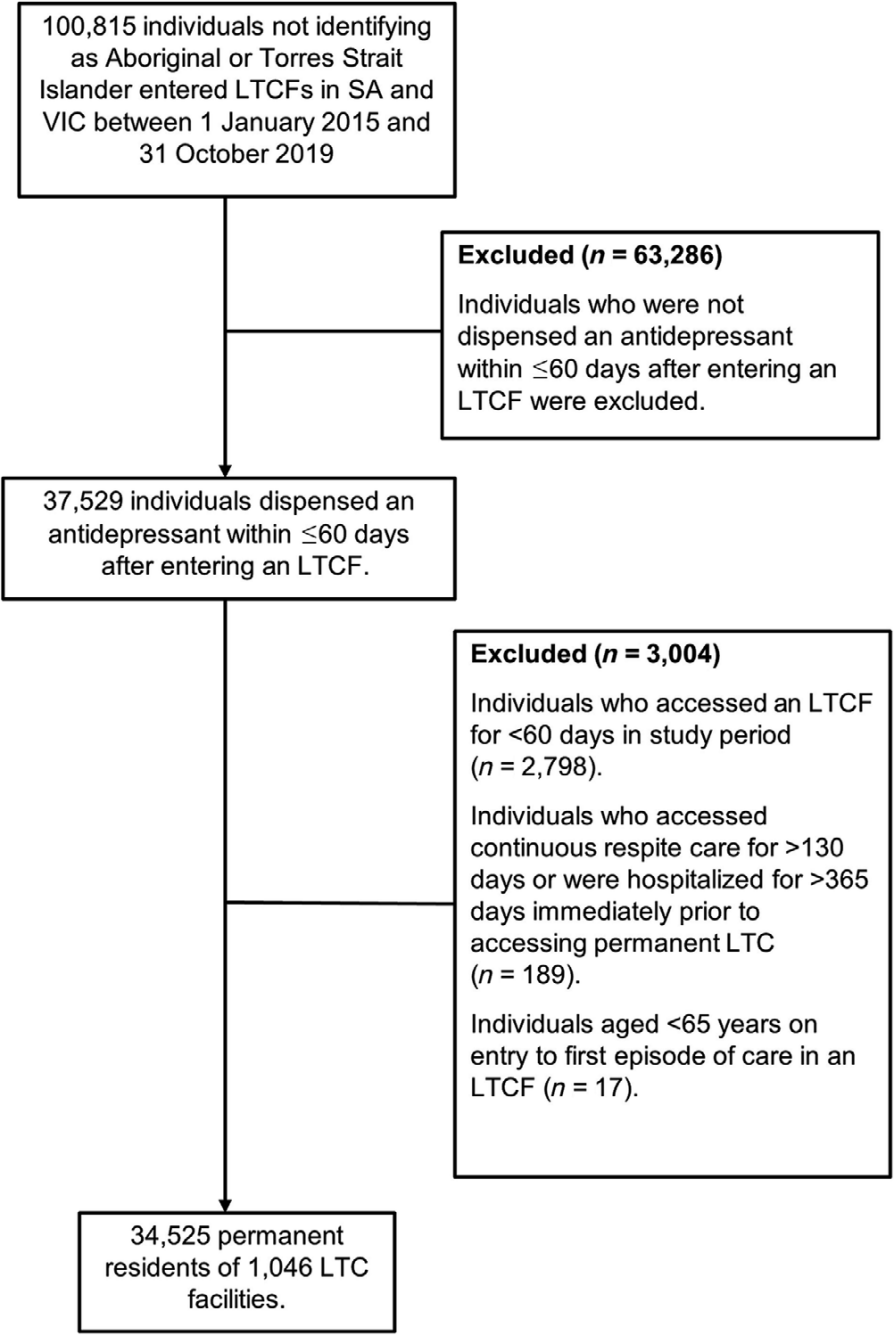
Flow chart for study cohort selection. LTC, long‐term care; LTCF, long‐term care facility; SA, South Australia; VIC, Victoria.

Non‐Indigenous individuals who entered permanent residential aged care for the first time in SA and VIC during 2015–2019, were aged 65–105 years on LTCF entry, dispensed an antidepressant between LTCF entry and ≤60 days afterwards, and accessed continuous long‐term care for ≥60 days were included (Figure 1). The final cohort included 34 525 individuals.

In Australia, LTCFs provide transition (i.e., short‐term care after a hospitalization), respite (temporary) or permanent residential care.^18^ To determine an LTCF entry date, consecutive records of an individuals' transition, respite and permanent long‐term care (i.e., ±1 day between exit and re‐entry) were linked to form LTCF episodes. Residents with prolonged respite stays (i.e., >130 days) were excluded.

Antidepressant use was ascertained using ATC codes N06A* (excluding clomipramine [N06AA04], bupropion [N06AX12], agomelatine [N06AX22], vortioxetine [N06AX26] and esketamine nasal spray [N06AX27]).^36^ Antidepressants were characterized as selective serotonin reuptake inhibitors (SSRIs), mirtazapine, serotonin and noradrenaline reuptake inhibitors (SNRIs), tricyclic antidepressants (TCAs), monoamine oxidase inhibitors (MAOIs) and ‘others’.

### 2.3 Outcomes of interest

The outcomes of interest were the care setting where antidepressants were initiated (i.e., community‐based, hospital or LTCF) and resident characteristics associated with initiation.

To identify the setting of initiation, hospital claims were screened to determine individuals with an overnight hospitalization between LTCF entry and ≤30 days prior. Consecutive hospital claims (±1 day) were linked to form hospitalization episodes and individuals discharged to a LTCF (±1 day) were identified. Pharmaceutical claims in the 120 days (approximately four antidepressant prescription durations for once daily antidepressants dispensed in four‐week quantities^37^, ^38^) before hospitalization (for those hospitalized) or LTCF entry (for those not hospitalized) were screened to determine initiation setting. Individuals dispensed an antidepressant during the 120 days prior to LTCF or hospitalization were considered initiated in a community‐based setting (existing users). In‐hospital initiation (new users) was defined by a first dispensing between the hospital admission and discharge dates or LTCF entry, whichever occurred last (<1% had overlapping hospital and LTCF dates). Initiation within LTCFs (new users) was defined by a first dispensing on or after the LTCF entry date, or where an individual was hospitalized prior, from the day after LTCF entry or hospital discharge, whichever occurred last (Supplementary Figure S1).

### 2.4 Covariates

Resident characteristics examined included age at LTCF entry, sex, country of birth, preferred language and marital status. A modified Rx‐Risk‐V Comorbidity Index score was determined using pharmaceutical claims 6 months prior to LTCF entry (excluding antidepressants).^39^ Depression was determined from aged care eligibility and LTCF entry assessments. Dementia was determined from aged care eligibility assessments, LTCF entry assessments and pharmaceutical claims for medicines used for dementia symptoms during 6 months before LTCF entry.^18^ Pharmaceutical claims in the 120 days before LTCF entry were used to determine number of unique medicines (excluding antidepressants), and recent use of antipsychotics (WHO ATC codes N05A*; excluding prochlorperazine [N05AB04] and lithium [N05AN01], or benzodiazepines and zopiclone [N05BA*, N05AE*, N05CD*, N05CF01]) in the 120 days before hospital or LTCF entry.^18^, ^36^

LTCF ownership (government, not‐for‐profit, private), Australian state (SA, VIC) and remoteness (major cities, outside major cities) were determined. Facility volume was determined as the number of residents in the LTCF on 30 June for the year of LTCF entry. If facility volume was zero on 30 June (<1% individuals' facility volume), due to resident turnover or change in LTCF operations, the resident volume in the year closest to or following LTCF entry was selected.

### 2.5 Statistical analysis

**FIGURE 2:**
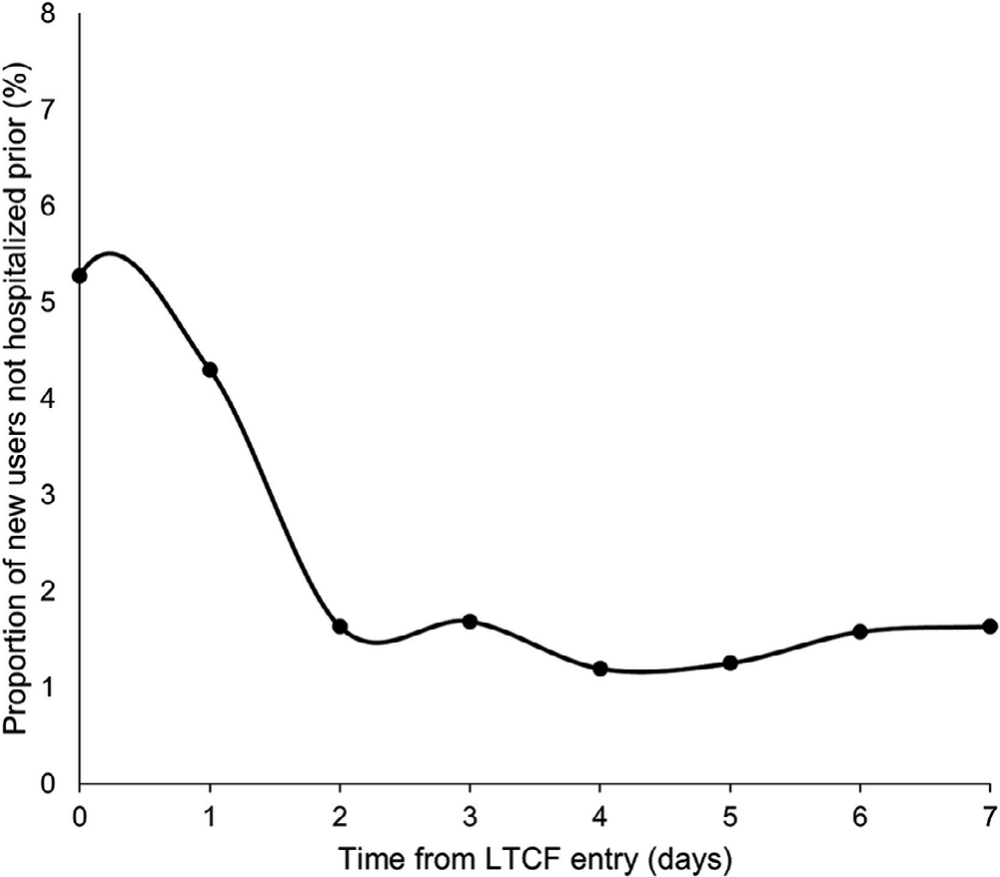
First day of antidepressant initiation relative to LTCF entry among individuals who were not hospitalized or taking an antidepressant before entering long‐term care (*n* = 1839). Day 0 is day of LTCF admission. LTCF, long‐term care facility.

Resident characteristics, setting of antidepressant initiation, antidepressant type, reason for hospitalizations preceding LTCF entry and length of hospital stay were summarized using descriptive statistics. Date of antidepressant initiation relative to LTCF entry and hospital discharge (for those hospitalized prior) were examined graphically (Figure 2, Supplementary Figure S2). Associations between resident characteristics and setting of antidepressant initiation were initially examined using bivariate multinomial logistic regression models using the covariates listed above, then variables associated with initiation setting (cut‐off *P*‐values ≤0.2, *n* = 10 covariates) were examined and adjusted for in a backward stepwise multinomial logistic regression model. Model goodness‐of‐fit was assessed using the Hosmer–Lemeshow test. Model assumptions were assessed and met. A complete case analysis was carried out (*n* = 753, 2.2% missing data). In a sensitivity analysis, facility characteristics were also evaluated to determine if they were associated with antidepressant initiation in an LTCF. Data were analysed using SAS, version 9.4 (SAS Institute Inc., Cary, NC).

### 2.6 Ethical considerations

Ethical approvals were obtained from the University of South Australia (200489), AIHW (EO2022/4/1376) and SA Department for Health and Wellbeing (HREC/18/SAH/90) Human Research Ethics Committees.

## 3 RESULTS

This study included 34 525 individuals from 1046 LTCFs (Figure 1). There were 64.7% (*n* = 22 346) residents who were female, 48.4% (*n* = 16 711) were aged ≥85 years, 75.6% (*n* = 26 086) had a comorbidity score of 3–8 and 48.9% (*n* = 16 888) were living with dementia (Table 1). Overall, 72.8% (*n* = 25 136) of residents had depression recorded in aged care assessments and 34.7% (*n* = 11 968) recently used another psychotropic (Supplementary Table S1). Residents primarily accessed privately owned (40.1%, *n* = 13 841) and not‐for‐profit (29.2%, *n* = 10 095) LTCFs, and lived in major cities (70.8%, *n* = 24 459). There were 58.8% (*n* = 20 304) residents hospitalized in the month prior to LTCF entry, of which 89.3% (*n* = 18 126) discharged directly to an LTCF.

**TABLE 1:** Study cohort characteristics, overall and by setting of antidepressant initiation.

Characteristics	Overall	Community^a^	Hospital^b^	LTCF^c^
	*n* (%)	*n* (%)	*n* (%)	*n* (%)
**Number of residents**	34 525 (100.0)	27 160 (78.7)	2552 (7.4)	4813 (13.9)
**Female sex**	22 346 (64.7)	18 054 (66.5)	1370 (53.7)	2922 (60.7)
**Age at LTCF entry**				
65–74 years	4685 (13.6)	3811 (14.0)	387 (15.2)	487 (10.1)
75–84 years	13 129 (38.0)	10 505 (38.7)	1005 (39.4)	1619 (33.6)
85–89 years	9707 (28.1)	7591 (28.0)	699 (27.4)	1417 (29.4)
≥90 years	7004 (20.3)	5253 (19.3)	461 (18.1)	1290 (26.8)
**Born in Australia**	22 352 (64.7)	17 769 (65.6)	1523 (59.7)	3060 (63.6)
**Primary language spoken is English**	30 132 (87.3)	23 768 (87.5)	2178 (85.3)	4186 (87.0)
**Comorbidity score on LTCF entry** ^**d**^				
0–2	4900 (14.2)	3211 (11.8)	526 (20.6)	1163 (24.2)
3–5	14 237 (41.2)	11 270 (41.5)	1009 (39.5)	1958 (40.7)
6–8	11 849 (34.3)	9702 (35.7)	792 (31.0)	1355 (28.2)
9+	3539 (10.3)	2977 (11.0)	225 (8.8)	337 (7.0)
**Dementia diagnosis on LTCF entry**	16 888 (48.9)	13 374 (49.2)	1036 (40.6)	2478 (51.5)
**Depression diagnosis on LTCF entry**	25 136 (72.8)	20 328 (74.9)	1757 (68.9)	3051 (63.4)
**Concession card holder**	32 612 (94.5)	25 731 (94.7)	2401 (94.1)	4480 (93.1)
**Marital status**				
Widowed	15 652 (45.3)	12 331 (45.4)	1066 (41.8)	2255 (46.9)
Married/de facto	13 466 (39.0)	10 725 (39.5)	958 (37.5)	1783 (37.1)
Divorced/separated	2959 (8.6)	2317 (8.5)	243 (9.5)	399 (8.3)
Never married	1926 (5.6)	1411 (5.2)	216 (8.5)	299 (6.2)
**State of residence at LTCF entry**				
VIC	25 755 (74.6)	20 222 (74.5)	2000 (78.4)	3533 (73.4)
SA	8770 (25.4)	6938 (25.5)	552 (21.6)	1280 (26.6)
**Remoteness and ownership of LTCF**				
*Major city:*				
Private	13 841 (40.1)	10 770 (39.7)	1105 (43.3)	1966 (40.8)
Not‐for‐profit	10 095 (29.2)	7909 (29.1)	697 (27.3)	1489 (30.9)
Government	523 (1.5)	392 (1.4)	52 (2.0)	79 (1.6)
*Outside major city:*				
Private	2814 (8.2)	2272 (8.4)	192 (7.5)	350 (7.3)
Not‐for‐profit	4616 (13.4)	3805 (14.0)	271 (10.6)	540 (11.2)
Government	2479 (7.2)	1892 (7.0)	224 (8.8)	363 (7.5)
**Individuals discharged from hospital to LTCF**	18 126 (52.5)	13 051 (48.1)	2375 (93.1)	2700 (56.1)
**Hospitalized between LTCF entry and ≤30 days prior**				
≥1 hospitalization	20 304 (58.8)	14 778 (54.4)	2552 (100.0)	2974 (61.8)
≥2 hospitalizations	1797 (5.2)	1341 (4.9)	205 (8.0)	251 (5.2)
**Length of hospital stay (days), median [IQR]**	26 (13–46)	23 (11–42)	45 (25–71)	28 (14–49)
**Use of other psychotropics in the 120 days before hospital or LTCF admission**				
Any psychotropic^d^	11 968 (34.7)	10 088 (37.1)	614 (24.1)	1266 (26.3)
Antipsychotic	4074 (11.8)	3596 (13.2)	147 (5.8)	331 (6.9)
BZD or zopiclone	7894 (22.9)	6492 (23.9)	467 (18.3)	935 (19.4)
**Number of unique medicines dispensed in the 120 days before LTCF admission** ^**d**^				
0–5	11 005 (31.9)	7955 (29.3)	996 (39.0)	2054 (42.7)
6–10	14 045 (40.7)	11 379 (41.9)	910 (35.7)	1756 (36.5)
11–16	7558 (21.9)	6220 (22.9)	515 (20.2)	823 (17.1)
17+	1917 (5.6)	1606 (5.9)	131 (5.1)	180 (3.7)

Abbreviations: BZD, benzodiazepine; IQR, interquartile range; LTCF, long‐term care facility, SA, South Australia; VIC, Victoria. Individuals missing information: *n* = 97 (0.3%) country of birth; *n* = 221 (0.6%) primary language; *n* = 2457 (7.1%) aged care eligibility assessment; *n* = 53 (0.2%) entry to care assessment; *n* = 157 (0.5%) remoteness and ownership; *n* = 522 (1.5%) marital status.

^a^ Defined as antidepressant use in the 4 months (120 days) prior to hospital or LTCF admission (existing users).

^b^ Antidepressant initiated in hospital prior to LTCF entry (new users).

^c^ Antidepressant initiated in the LTCF (new users).

^d^ Type of psychotropic used in the 120 days before hospitalization or LTCF entry determined from the earliest dispensing during the 120 days (excludes antidepressants).

Most residents (78.7%, *n* = 27 160) receiving an antidepressant during transition to LTCF initiated treatment prior to entry, while 7.4% (*n* = 2552) initiated in hospitals and 13.9% (*n* = 4813) in LTCFs (Table 1). There were 31.8% (*n* = 1529) of LTCF initiators who commenced within 14 days of LTCF entry, including 9.6% (*n* = 176) of 1839 new users who were not hospitalized and commenced on the day of, or following, LTCF entry (Figure 2, Supplementary Figure S3). The overall median length of stay for hospitalizations closest to LTCF entry was 26 days (IQR 13–46), and 45 days (IQR 25–71) for those where antidepressants were initiated. Principal diagnoses for hospitalizations are presented in Supplementary Table S2. Mirtazapine was the most common individual antidepressant initiated across all settings, and the leading class of antidepressants initiated in hospitals (44.8%, *n* = 1143) (Figure 4, Supplementary Table S3). SSRIs were the most frequently used class overall (47.8%, *n* = 16 516) and among community‐based initiators (49.1%, *n* = 13 325) and LTCF initiators (44.4%, *n* = 2136). SNRIs and TCAs were more frequently initiated in community‐based settings (16.1%, *n* = 4379; 13.0%, *n* = 3527, respectively) than hospitals (9.0%, *n* = 229; 5.6%, *n* = 144) and LTCFs (7.7%, *n* = 370; 8.7%, *n* = 421). There were negligible differences in antidepressants initiated annually (Supplementary Figure S4).

**FIGURE 3:**
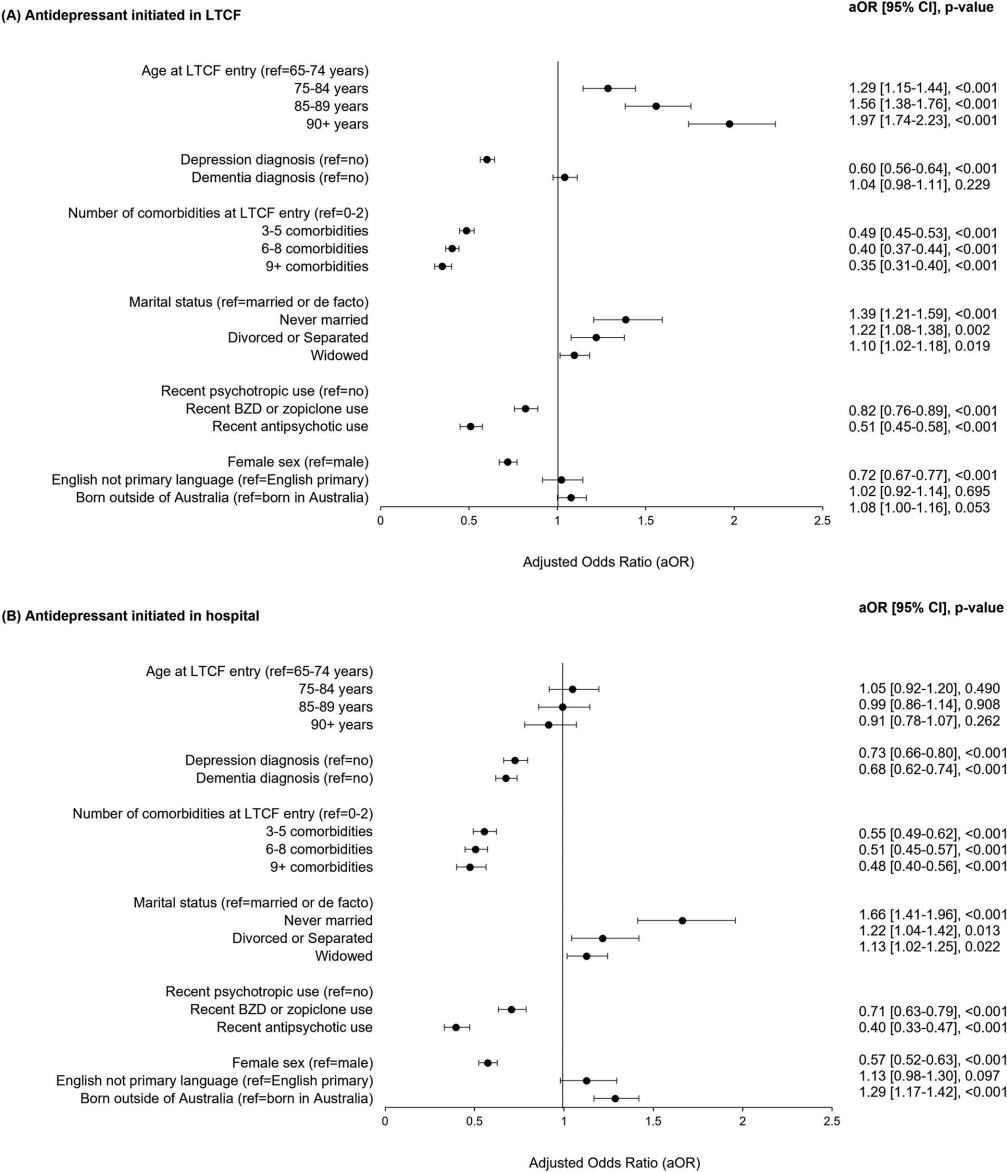
Adjusted odds ratio with 95% confidence intervals for multivariate multinomial logistic regression models evaluating resident characteristics associated with antidepressant initiation in (A) LTCF and (B) hospital. Reference group = individuals initiating prior to entry (i.e., in community). BZD, benzodiazepine; LTCF, long‐term care facility.

Figure 3 presents adjusted associations between resident characteristics and setting of antidepressant initiation. Individuals with a higher comorbidity score (i.e., ≥9) were 65% and 52% more likely to start an antidepressant prior to entry, compared to a LTCF or hospital (aOR = 0.35, 95% CI 0.31–0.40; aOR = 0.48, 95% CI 0.40–0.56). Females and those recently using a psychotropic were less likely to initiate antidepressants in LTCFs and hospitals. Residents with dementia were less likely to initiate in hospital compared to prior to LTCF entry (aOR = 0.68, 95% CI 0.62–0.74). Older residents were more likely to start an antidepressant in the LTCF compared to prior to entry (aOR = 1.97, 95% CI 1.74–2.23 for residents aged ≥90 years *vs*. 65–74 years).

**FIGURE 4:**
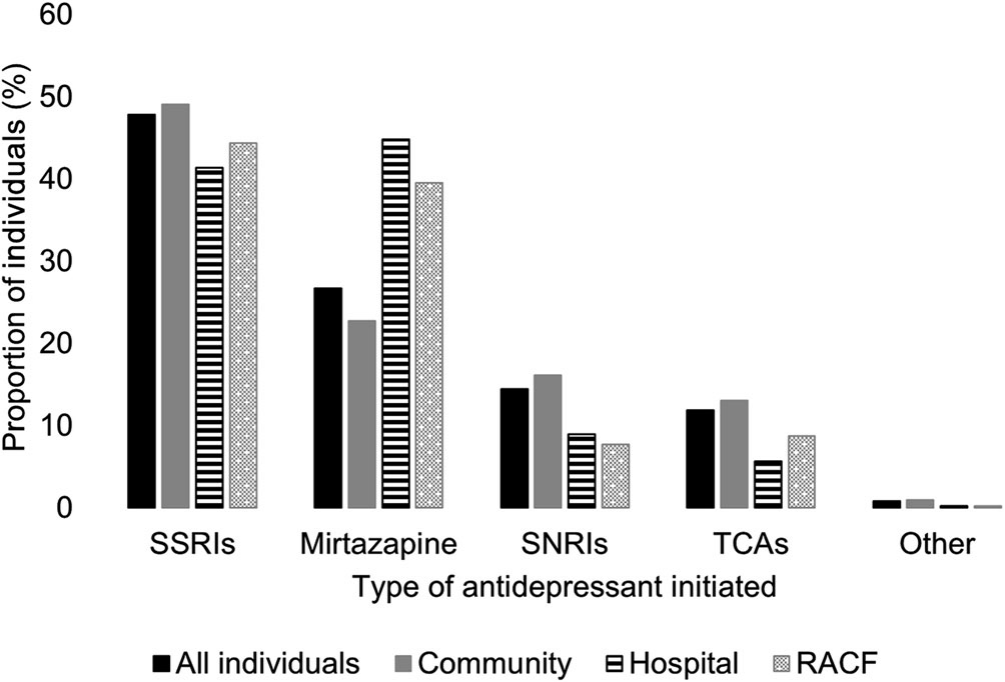
Proportion of individuals who initiated an antidepressant, by antidepressant class and setting of initiation. Monoamine oxidase inhibitors excluded due to low counts. All antidepressant types were counted for *n* = 560 (1.6%) of people who initiated >1 antidepressant on the same date. Other, other antidepressants; RACF, residential aged care facility (interchangeable term for long‐term care facility); SNRIs, serotonin and noradrenaline reuptake inhibitors; SSRIs, selective serotonin reuptake inhibitors; TCAs, tricyclic antidepressants.

Supplementary Figure S5 shows individuals living outside of major cities in privately owned (aOR = 0.83, 95% CI 0.73–0.94) or not‐for‐profit LTCFs (aOR = 0.74, 95% CI 0.66–0.82) were less likely to commence antidepressant therapy within the LTCF. Negligible change in aORs for resident characteristics were observed when facility factors were included in the model. Supplementary Table S4 presents global *P*‐values for covariates included in the model.

## 4 DISCUSSION

This population‐based study examined the care setting of antidepressant initiation among 34 525 individuals transitioning to LTCFs. Although 78.7% of our cohort were existing antidepressant users, 7.4% commenced the antidepressant in hospital before LTCF entry, and 13.9% after entry. Older age and being single were associated with antidepressant initiation in LTCFs or hospitals, compared to commencing therapy prior to LTCF entry (i.e., in community‐based care settings). In contrast, residents who were female, with a greater comorbidity burden, recorded depression diagnosis, and who had recently used another psychotropic had lower odds of initiating the antidepressant in hospital or a LTCF, compared to before entering LTCFs. Our findings suggest that efforts to optimize antidepressant use within LTCFs, used by 60% of residents annually,^23^ should also consider the contribution of hospitals and community‐based care settings, where antidepressants are also initiated.

Recent hospitalization is a known predictor for antidepressant initiation in older people.^11^, ^40^ This is the first study to examine antidepressant initiation during hospitalizations that immediately precede LTCF entry, and associated factors. The median length of hospital stay among people initiating antidepressants in hospital was 1.5 months (45 days). Individuals initiating antidepressants had longer hospital stays which highlights the potential psychological impacts of hospitalization on older people. There is also potential for antidepressant adverse effects, such as restlessness, sedation and nausea, to exacerbate hospital‐associated deconditioning and lead to further physical and psychological harms.^41^ Antidepressants are the leading cause of hospitalizations due to administration errors of psychotropic drugs (48.5%) in Australia, and contribute to 11% of drug‐related hospitalizations among older people with cognitive impairment in Sweden.^42^, ^43^ Transitions of care also place individuals at risk of medicines‐related harm due to problems with information sharing and clinical handover, and few residents receive a medicines review at LTCF entry.^10^, ^32^, ^44^ Optimizing the use and uptake of digital health advances in the community, LTCFs and hospitals to improve timely information sharing between care settings is an important avenue for ongoing development.^44^ Interventions targeting antidepressant use in LTCFs should consider the use of antidepressants among community‐dwelling older people and those in hospital, and could include expanding quality indicators and psychotropic stewardship programmes to monitor antidepressant use across health and aged care settings.

More than four in five new antidepressant users were first dispensed mirtazapine or an SSRI, which are the most common antidepressants used in Australian LTCFs.^23^, ^45^ SSRIs, mirtazapine and some SNRIs are recommended first line therapy for pharmacological management of moderate to severe major depression, and some SSRIs (e.g., citalopram) may also be used for changed behaviours in dementia in Australia.^46^, ^47^, ^48^ However, for both mild to moderate major depression and changed behaviours in dementia, antidepressants are only recommended in conjunction with, and after initially trialling, non‐pharmacological strategies,^47^, ^48^, ^49^ which are often underutilized in LTCFs.^46^ Systematic reviews found insufficent evidence to support the use of antidepressants in people with dementia (49% of individuals in this study), and current practice guidelines do not support mirtazapine use in this cohort.^46^, ^50^, ^51^ Despite this, our study found mirtazapine, a tetracyclic antidepressant with sedative and weight‐gain properties, was the most commonly used individual antidepressant across all care settings and its initiation in hospitals surpassed all SSRIs collectively. Further, mirtazapine use almost doubled among new users (44.8% initiated in hospital and 39.5% in LTCFs), compared to those who initiated treatment prior to entry (22.7%). Although some antidepressants are indicated for conditions other than depression (e.g., duloxetine can be used for neuropathic pain), mirtazapine is only indicated for treating moderate to severe major depression. In this study, 36.6% of LTCF initiators, and 31.1% of hospital initiators did not have depression (of any severity) recorded. This aligns with a study in Belgian LTCFs in which only 21.2% of antidepressant use was for conditions other than depression (i.e., insomnia, anxiety or pain), and only 8% of mirtazapine use was for peripheral neuropathy in Denmark.^52^, ^53^ It is therefore likely mirtazapine is being used off‐label, particularly for people in hospital or entering LTCFs, who are often frail, living with dementia and may be experiencing delirium, changed behaviours, insomnia, pain, loneliness, sadness or loss. It is possible that these other indications, and the likely over‐reliance on antidepressants as the primary treatment modality (less than 3% of residents of Australian LTCFs access government‐subsidized mental health services^54^), may contribute to the high use of these medicines among older people. Our findings highlight the need to optimize evidence‐based management of mental health among older people hospitalized and entering LTCFs, including improving access to non‐pharmacological therapies, to uphold the safe and effective use of antidepressants. This study emphasizes the importance of ongoing cross‐sectoral monitoring for depression and antidepressant use in this setting. Moreover, few studies have examined safety and effectiveness of mirtazapine use in older people in LTCFs, and this remains an important research priority.^55^

On balance, factors associated with antidepressant initiation were similar between LTCFs and hospitals and were consistent with known risk factors for antidepressant use.^11^, ^19^, ^52^ For example, women, people recently using other psychotropics or with high comorbidity burden were more likely to be existing antidepressant users. Marital status did not influence antidepressant initiation in older people in Denmark;^52^ however, in this study residents without a spouse (i.e., never married, widowed or divorced/separated) had up to 66% higher odds of starting an antidepressant around LTCF entry than existing users. This suggests marital status may be more associated with antidepressant use among those living in LTCFs specifically, where up to 55% of residents experience loneliness.^56^ In the present study, residents taking an antidepressant with depression (of any severity) recorded in their care assessments, were more likely to be existing users, indicating those with diagnosed depression are more likely to begin treatment earlier. This study found residents were more likely to commence treatment prior to LTCF entry if they resided in a not‐for‐profit or privately owned LTCF outside of a major city, consistent with another study showing antidepressant utilization in LTCFs is lower outside major cities.^16^ The integration of pharmacists to routine care provision in LTCFs is a promising opportunity to create meaningful change to optimize resident outcomes, expanding regular review of residents and monitoring of medicines use and safety, including for antidepressants.^57^ However, this should be accompanied with national policy improvements, including expansion of Australia's quality indicator programme, increased education, further research and continual monitoring for the effect of new interventions. Moreover, this study finds most individuals using antidepressants during entry to an LTCF had commenced the medicine before LTCF entry, particularly in community‐based settings. Future quality improvement initiatives to optimize antidepressant use in this setting should also target antidepressant initiation and discontinuation within primary care.

### 4.1 Strengths and limitations

This is the first study to directly examine the care setting of antidepressant initiation for individuals entering LTCFs and associated factors. Individuals from LTCFs in two Australian states (~36% of residents nationwide^23^) were included and hence findings are likely generalizable to the broader LTCF population. Limitations include possible misattribution of antidepressant initiation between hospitals and LTCFs as PBS‐subsidized antidepressants were only supplied at hospital discharge and inpatient use is not captured within ROSA. Antidepressants initiated before hospitalization or LTCF entry were considered commenced in ‘community‐based settings’; however, we did not screen more than 4 months prior to identify the specific setting of initiation for these users. Only factors included in our dataset could be assessed in regression models, hence some important factors not captured in ROSA, such as depression severity, indication(s) for antidepressant use (e.g., pain) and dose, could not be included, and thus appropriateness of use could not be determined. Examining the dose of antidepressants used would have provided important context to the indication (such as lower doses of TCAs for pain management), likely effectiveness of the medicine for treating depression, risk of harm associated with sedative properties and potential off‐label use (such as low‐dose mirtazapine for insomnia).^52^, ^53^, ^55^, ^58^ Any analysis of Indigenous individuals' records require leadership from Indigenous individuals and specific Indigenous governance and ethics approvals, which were not part of this study.^59^ More research is needed to determine persistence with antidepressant therapy post‐LTCF admission, discontinuation as residents approach end‐of‐life, and safety of mirtazapine as the clearly preferred agent within this cohort.

## 5 CONCLUSION

Individuals who transition to permanent care in a LTCF often have complex transitions, during which antidepressants may be initiated. This study highlights the importance of understanding the setting and recency of antidepressant initiation, indication for use and subsequent monitoring plans when evaluating antidepressant utilization in LTCFs. With one‐third of residents commencing antidepressant therapy in LTCFs starting therapy within 2 weeks of entry, there are considerable concerns for the safe and effective use of antidepressants used among this cohort. Future quality improvement and monitoring activities to optimize antidepressant use in LTCFs should also consider hospital and primary care settings.

## AUTHOR CONTRIBUTIONS

JKS, GAH, MCI, DR and MC conceived the study. GAH and JKS drafted the study protocol which was reviewed by all investigators. CL prepared the data for analysis. GAH analysed the data. GAH drafted the manuscript. All authors critically reviewed, read and approved the final manuscript.

## CONFLICT OF INTEREST STATEMENT

J.K.S. is a non‐executive director of Southern Cross Care SA, NT, VIC (an aged care provider organization). There are no other competing interests to declare.

## Supporting information


**Supplementary Figure S1.** Definitions for determining setting of antidepressant initiation among (A) individuals entering LTCF without prior hospitalization; and (B) individuals entering LTCF with prior hospitalization who took an antidepressant in the first 60 days of LTCF entry.
**Supplementary Figure S2.** Day of antidepressant initiation among new antidepressant users who were hospitalized prior to long‐term care facility entry (*n* = 5526), relative to hospital discharge.
**Supplementary Figure S3.** Day of antidepressant initiation within 2 weeks of LTCF entry among individuals who initiated in an LTCF (*n* = 4813).
**Supplementary Figure S4.** Annual antidepressant initiation, stratified by year (2015–2019).
**Supplementary Figure S5.** Adjusted odds ratio with 95% confidence intervals for multivariate logistic regression model examining resident and facility characteristics associated with antidepressant initiation in long‐term care facilities.
**Supplementary Table S1.** Other psychotropic medicines dispensed 120 days before hospital admission or LTCF entry.
**Supplementary Table S2.** Primary reason for hospitalization for (i) individuals with hospitalization(s) in long‐term care (*n* = 20 304) and (ii) individuals initiating an antidepressant in hospital (*n* = 2552).
**Supplementary Table S3.** Class of antidepressant initiated, overall and by setting of initiation.
**Supplementary Table S4.** Global *P*‐values (from Type III analyses of effects) for individual covariates included in the primary multivariate multinomial logistic regression model and sensitivity analysis examining addition of facility factors.

## Data Availability

Data may be obtained from a third party and are not publicly available. The data for this study were obtained from the Australian Institute of Health and Welfare, Australian Government Department of Health and South Australia and Victoria state health authorities and integrated by the Australian Institute of Health and Welfare, the Centre for Victorian Data Linkage and SA NT DataLink. These data were made available to the researchers under ethical, governance and confidentiality agreements that do not allow public sharing.
