# Supplementary material for: Setting of initiation and factors associated with antidepressant use on entry to long‐term care facilities
Source: Br J Clin Pharmacol. 2025 Jan 31;91(6):1749–59. doi: 10.1111/bcp.16403 (PMC12122125; doi:10.1111/bcp.16403)
Supplement: Supplementary file 1 — Supplementary Figure S1. Definitions for determining setting of antidepressant initiation among (A) individuals entering LTCF without prior hospitalization; and (B) individuals entering LTCF with prior hospitalization who took an antidepressant in the first 60 days of LTCF entry. Supplementary Figure S2. Day of antidepressant initiation among new antidepressant users who were hospitalized prior to long‐term care facility entry (n = 5526), relative to hospital discharge. Supplementary Figure S3. Day of antidepressant initiation within 2 weeks of LTCF entry among individuals who initiated in an LTCF (n = 4813). Supplementary Figure S4. Annual antidepressant initiation, stratified by year (2015–2019). Supplementary Figure S5. Adjusted odds ratio with 95% confidence intervals for multivariate logistic regression model examining resident and facility characteristics associated with antidepressant initiation in long‐term care facilities. Supplementary Table S1. Other psychotropic medicines dispensed 120 days before hospital admission or LTCF entry. Supplementary Table S2. Primary reason for hospitalization for (i) individuals with hospitalization(s) in long‐term care (n = 20 304) and (ii) individuals initiating an antidepressant in hospital (n = 2552). Supplementary Table S3. Class of antidepressant initiated, overall and by setting of initiation. Supplementary Table S4. Global P‐values (from Type III analyses of effects) for individual covariates included in the primary multivariate multinomial logistic regression model and sensitivity analysis examining addition of facility factors. [file BCP-91-1749-s001.pdf]

Setting of initiation and factors associated with antidepressant use on entry to long-term care facilities

Georgina A Hughes BPharm(Hons)[a,b], Maria C Inacio PhD[b,c], Debra Rowett BPharm[a,d], Catherine Lang BPhys(Hons)[b], Robert N Jorissen PhD[b,c], Megan Corlis BA(AppSci)[e], Janet K Sluggett PhD[b,c]

a) University of South Australia, UniSA Clinical & Health Sciences, Adelaide, South Australia, Australia
b) Registry of Senior Australians (ROSA), South Australian Health & Medical Research Institute, Adelaide, South Australia, Australia
c) University of South Australia, UniSA Allied Health & Human Performance, Adelaide, South Australia, Australia
d) Drug and Therapeutics Information Service, Southern Adelaide Local Health Network, Adelaide, South Australia, Australia
e) Australian Nursing & Midwifery Federation SA Branch, Adelaide, South Australia, Australia

Corresponding author

Ms. Georgina A Hughes [a,b]

a) University of South Australia, UniSA Clinical & Health Sciences, Adelaide, South Australia, Australia
b) Registry of Senior Australians (ROSA), South Australian Health & Medical Research Institute, Adelaide, South Australia, Australia

Email: georgina.hughes@mymail.unisa.edu.au

SUPPLEMENTARY INFORMATION

Supplementary Figure S1. Definitions for determining setting of antidepressant initiation among A) individuals entering LTCF without prior hospitalization; and B) individuals entering LTCF with prior hospitalization who took an antidepressant in the first 60 days of LTCF entry.

Supplementary Figure S2. Day of antidepressant initiation among new antidepressant users who were hospitalized prior to long-term care facility entry (n=5,526), relative to hospital discharge.

Supplementary Figure S3. Day of antidepressant initiation within two weeks of LTCF entry among individuals who initiated in an LTCF (n=4,813).

Supplementary Figure S4. Annual antidepressant initiation, stratified by year (2015-2019).

Supplementary Figure S5. Adjusted odds ratio with 95% confidence intervals for multivariate logistic regression model examining resident and facility characteristics associated with antidepressant initiation in long-term care facilities.

Supplementary Table S1. Other psychotropic medicines dispensed 120 days before hospital admission or LTCF entry.

Supplementary Table S2. Primary reason for hospitalization for (i) individuals with hospitalization(s) long-term care (n=20,304) and (ii) individuals initiating an antidepressant in hospital (n=2,552).

Supplementary Table S3. Class of antidepressant initiated, overall and by setting of initiation.

Supplementary Table S4. Global p-values (from Type III analyses of effects) for individual covariates included in the primary multivariate multinomial logistic regression model and sensitivity analysis examining addition of facility factors.

Supplementary Figure S1. Definitions for determining setting of antidepressant initiation among A) individuals entering LTCF without prior hospitalization; and B) individuals entering LTCF with prior hospitalization who took an antidepressant in the first 60 days of LTCF entry.

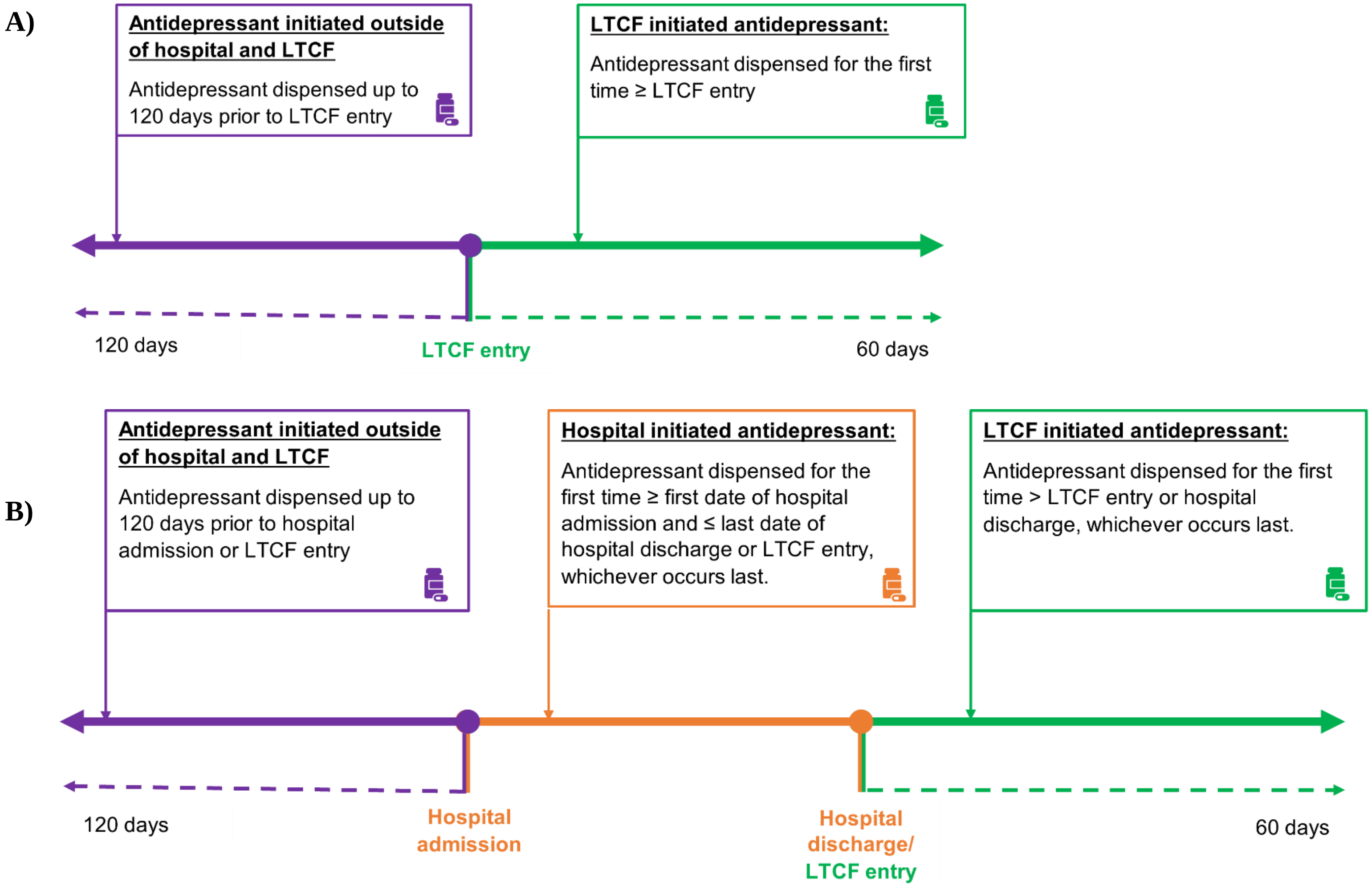

LTCF, long-term care facility.

Supplementary Figure S2. Day of antidepressant initiation among new antidepressant users who were hospitalized prior to long-term care facility entry (n=5,526), relative to hospital discharge.

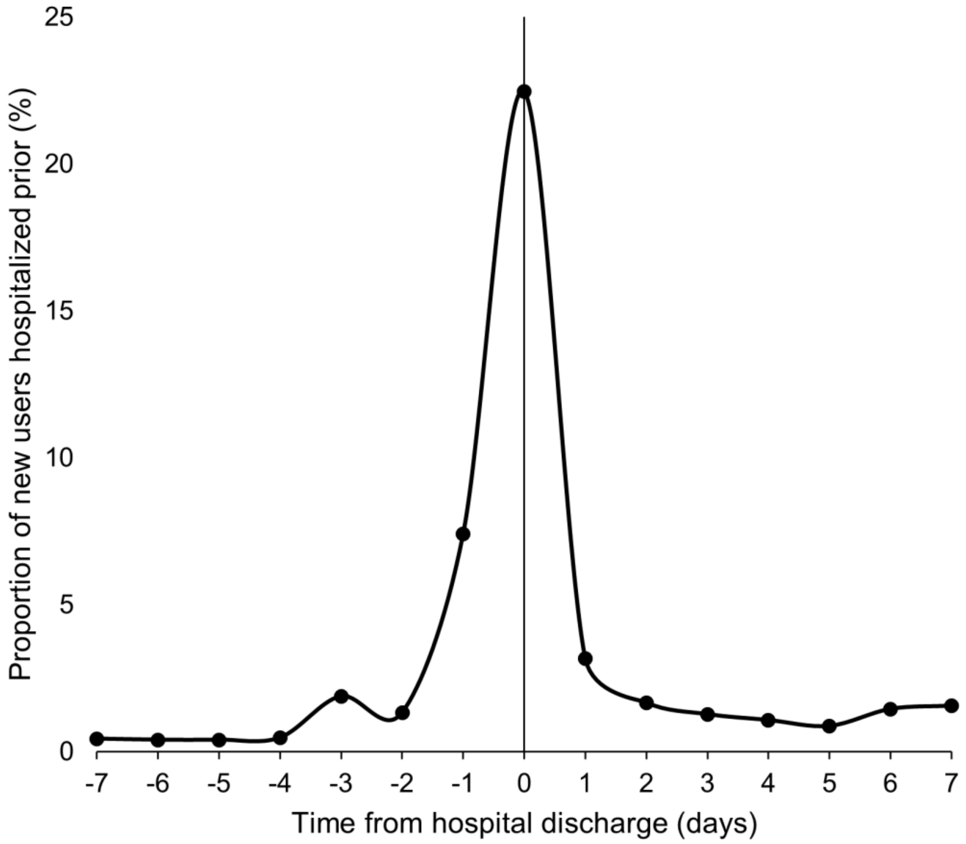

Time from hospital discharge is 0 days when antidepressant initiated on date of hospital discharge.

Supplementary Figure S3. Day of antidepressant initiation within two weeks of LTCF entry among individuals who initiated in an LTCF (n=4,813).

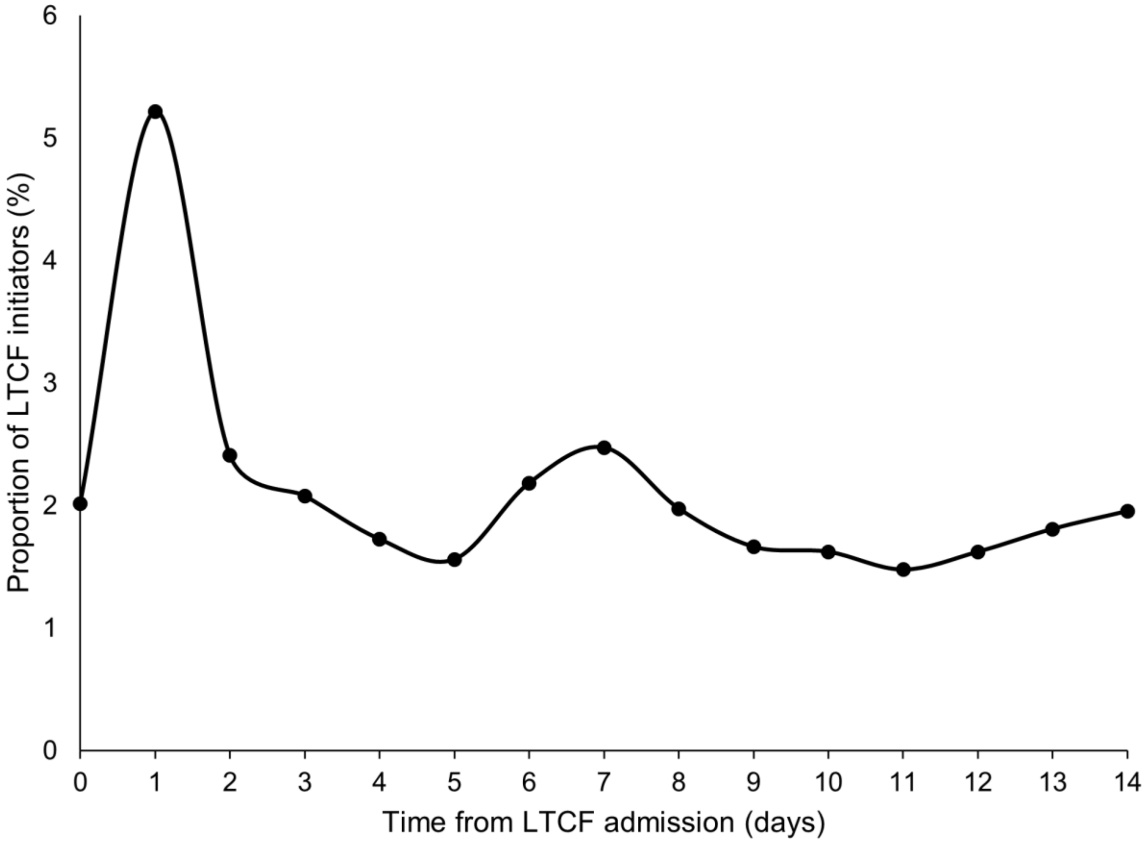

Day 0 is day of LTCF admission. LTCF, long-term care facility.

Supplementary Figure S4. Annual antidepressant initiation, stratified by year (2015-2019).

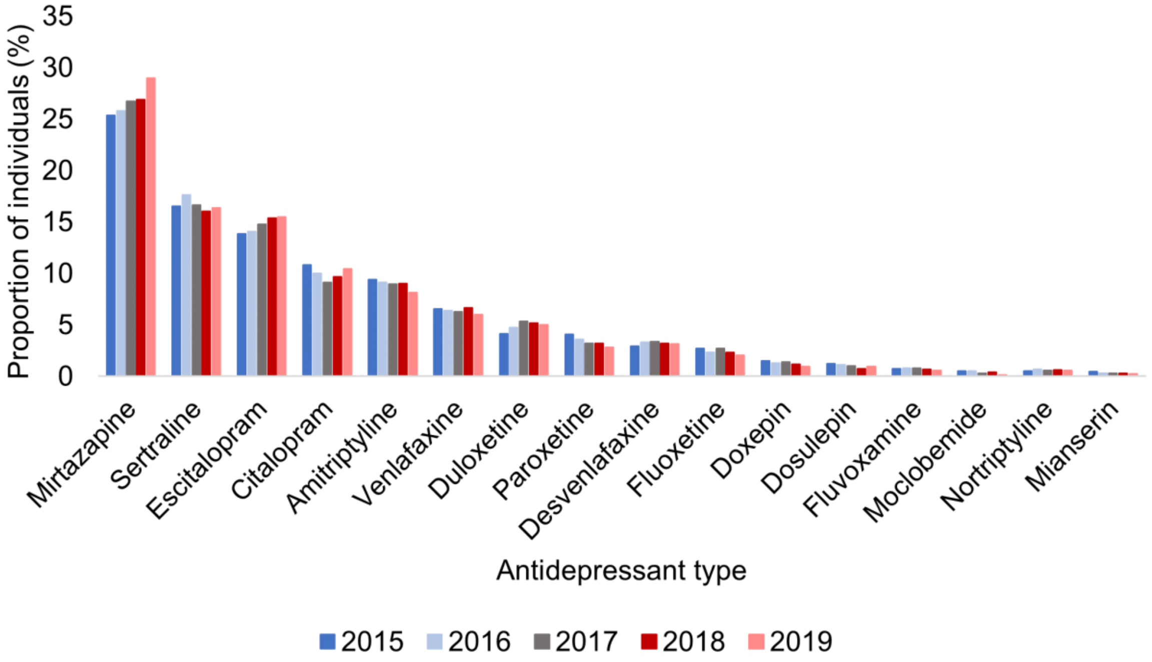

Monoamine oxidase inhibitors (tranylcypromine and phenelzine), reboxetine and imipramine not shown due to low counts. Type of antidepressant initiated was determined from the first antidepressant dispensing identified (i.e., during 120 days before LTCF or hospitalization for residents initiating in a community-based setting). Includes duplicates in numerator to determine proportions for n=560 (1.6%) people who initiated >1 antidepressant on the same date.

Supplementary Figure S5. Adjusted odds ratio with 95% confidence intervals for multivariate logistic regression model examining resident and facility characteristics associated with antidepressant initiation in long-term care facilities.

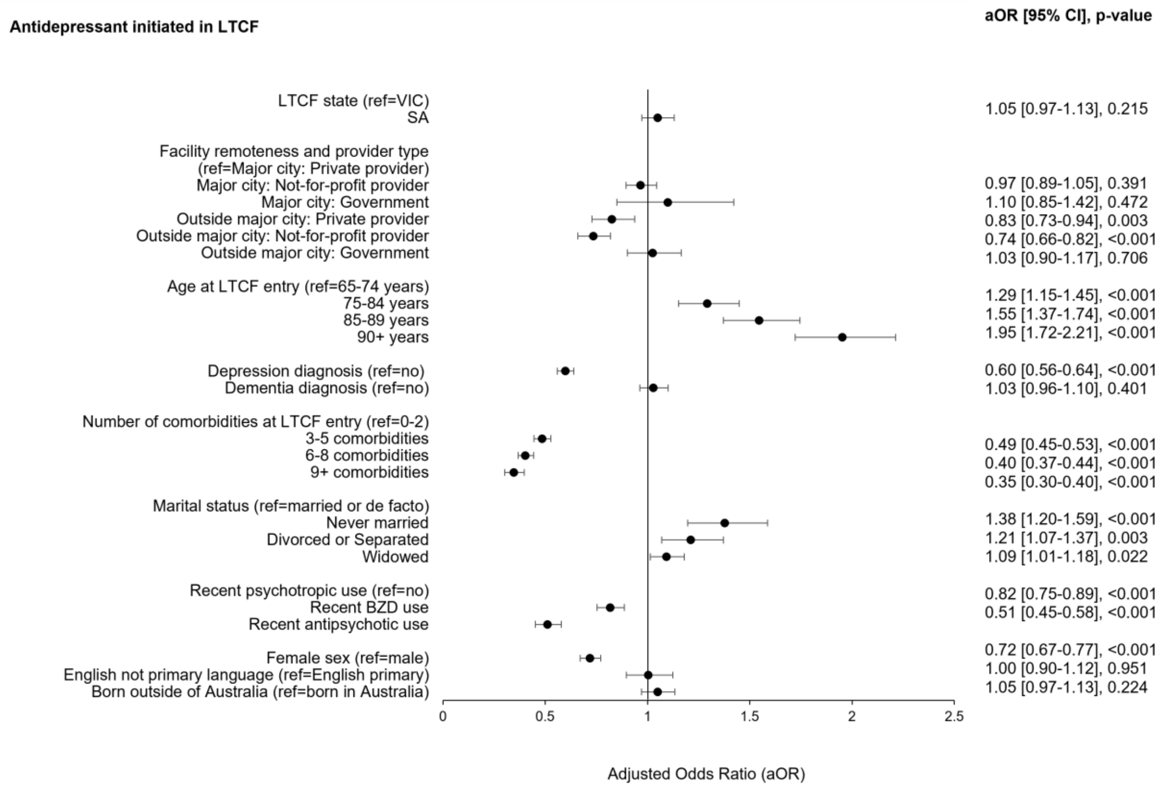

LTCF, long-term care facility, VIC, Victoria, SA, South Australia, BZD, benzodiazepine. Reference group = individuals initiating in community.

Supplementary Table S1. Other psychotropic medicines dispensed 120 days before hospital admission or LTCF entry.

**Group of individuals by setting of initiation**	**Recent use of a psychotropic medicine (120 days before hospital admission or LTCF entry) [n,%]**	**Top 6 psychotropic medicines dispensed 120 days before hospital admission or LTCF entry [n,%]**	
Overall cohort (n=34,525)	11 968 (34.7%)	Temazepam	3 499 (29.2)
		Oxazepam	2 199 (18.4)
		Risperidone	1 819 (15.2)
		Diazepam	1 584 (13.2)
		Quetiapine	961 (8.0)
		Olanzapine	779 (6.5)
Community (n=27,160)	10 088 (37.1%)	Temazepam	2 771 (27.5)
		Oxazepam	1 869 (18.5)
		Risperidone	1 595 (15.8)
		Diazepam	1 343 (13.3)
		Quetiapine	870 (8.6)
		Olanzapine	702 (7.0)
Hospital (n=2,552)	614 (24.1%)	Temazepam	232 (37.8)
		Oxazepam	113 (18.4)
		Diazepam	88 (14.3)
		Risperidone	60 (9.8)
		Quetiapine	38 (6.2)
		Nitrazepam	27 (4.4)
LTCF (n=4,813)	1 266 (26.3%)	Temazepam	496 (39.2)
		Oxazepam	217 (17.1)
		Risperidone	164 (13.0)
		Diazepam	153 (12.1)
		Olanzapine	53 (4.2)
		Quetiapine	53 (4.2)
		Nitrazepam	52 (4.1)

LTCF, long-term care facility. Most common psychotropic medicines recently used determined from the earliest dispensing in the screening period (120 days before entry to LTCF or hospital).

Supplementary Table S2. Primary reason for hospitalization for (i) individuals with hospitalization(s) long-term care (n=20,304) and (ii) individuals initiating an antidepressant in hospital (n=2,552).

**Primary reason for hospitalization[a]**	**Individuals with ≥1 hospitalisation preceding LTCF entry (n=20,304)**	**Individuals initiating an antidepressant in hospital (n=2,552)**
Certain infectious and parasitic diseases e.g., sepsis, gastroenteritis and colitis [A00-B99]	595 (2.9)	59 (2.3)
Neoplasms e.g., malignant neoplasms of lung, prostate [C00-D49]	610 (3.0)	85 (3.3)
Diseases of the blood and blood-forming organs and certain disorders involving the immune mechanism e.g., anaemia [D50-D89]	132 (0.7)	17 (0.7)
Endocrine, nutritional and metabolic disorders e.g., fluid, electrolyte and acid-base balance disorders, type 2 diabetes mellitus and complications, volume depletion, malnutrition [E00-E90]	478 (2.4)	65 (2.5)
Mental and behavioural disorders e.g., delirium and/or dementia, depressive episode, anxiety disorder [F00-F99]	2 912 (14.3)	377 (14.8)
Diseases of the nervous system e.g., Alzheimer's disease, Parkinson's disease, transient cerebral ischaemic attack, Lewy body disease [G00-G99]	1 092 (5.4)	140 (5.5)
Diseases of the eye and adnexa; diseases of the ear and mastoid process; and congenital malformations, deformations, and chromosomal abnormalities [H00-H59; H60-H95; Q00-Q99]	78 (0.4)	6 (0.2)
Diseases of the circulatory system e.g., heart failure, ischaemic stroke, myocardial infarction, arrhythmias, orthostatic hypotension [I00-I99]	2 970 (14.6)	565 (22.1)
Diseases of the respiratory system e.g., pneumonia, respiratory infection, chronic obstructive pulmonary disease, influenza [J00-J99]	1 738 (8.6)	160 (6.3)
Diseases of the digestive system e.g., intestinal obstructions, diverticulitis [K00-K95]	710 (3.5)	77 (3.0)
Diseases of the skin and subcutaneous tissue e.g., cellulitis, ulcer [L00-L99]	286 (1.4)	28 (1.1)
Diseases of the musculoskeletal system and connective tissue e.g., back pain, osteoporosis with fracture [M00-M99]	1 044 (5.1)	155 (6.1)

Diseases of the genitourinary system e.g., urinary tract infection, acute kidney failure, chronic kidney disease [N00-N99]	811 (4.0)	79 (3.1)
Symptoms, signs and abnormal clinical and laboratory findings, not elsewhere classified e.g., tendency to fall, malaise and fatigue, syncope and collapse, cognitive function/awareness [R00-R99]	2 521 (12.4)	242 (9.5)
Injury, poisoning and certain other consequences of external causes e.g., fractures, traumatic subdural haemorrhage, traumatic ischemia of muscle [S00-T88]	3 939 (19.4)	460 (18.0)
Factors influencing health status and contacts with health services e.g., rehabilitation, examination and observation following other accident, awaiting placement to residential aged care service, need for assistance at home and no other household member able to render care, respite care [Z00-Z99]	387 (1.9)	36 (1.4)

[a]Where consecutive claims for hospitalizations were linked to form a single hospitalization episode, the reason for hospitalization is for the first admission date only.

There were 55 (0.3%) individuals with ≥1 hospitalization who had awaiting placement to LTCF listed as the primary diagnosis for the hospitalization preceding LTCF entry (Z75.11).

LTCF, long-term care facility. Reason for hospitalization was the primary diagnosis of hospitalization closest to LTCF entry. There was <0.1% with missing information for reason for hospitalization.

Supplementary Table S3. Class of antidepressant initiated, overall and by setting of initiation.

**Antidepressant class**	**Overall (n=34,525) [n,%]**	**Community (n=27,160) [n,%]**	**Hospital (n=2,552) [n,%]**	**LTCF (n=4,813) [n,%]**
SSRIs	16 516 (47.8)	13 325 (49.1)	1 055 (41.3)	2 136 (44.4)
Mirtazapine	9 214 (26.7)	6 169 (22.7)	1 143 (44.8)	1 902 (39.5)
SNRIs	4 978 (14.4)	4 379 (16.1)	229 (9.0)	370 (7.7)
TCAs	4 092 (11.9)	3 527 (13.0)	144 (5.6)	421 (8.7)
Other	262 (0.8)	247 (0.9)	6 (0.2)	9 (0.2)
MAOIs	25 (0.1)	n/a	n/a	n/a

LTCF, long-term care facility, SSRIs, selective serotonin reuptake inhibitors, SNRIs, serotonin and noradrenaline reuptake inhibitors, TCAs, tricyclic antidepressants, Other, other antidepressants, MAOIs, monoamine oxidase inhibitors. MAOIs not shown due to low counts. All antidepressant types were counted for n=560 (1.6%) of people who initiated >1 antidepressant on the same date.

Supplementary Table S4. Global p-values (from Type III analyses of effects) for individual covariates included in the primary multivariate multinomial logistic regression model and sensitivity analysis examining addition of facility factors.

Covariate included in model	Global p-value (primary analysis)	Global p-value (sensitivity analysis)
Age at LTCF entry (ref=65-74 years)	<0.01	<0.01
Depression diagnosis (ref=no)	<0.01	<0.01
Dementia diagnosis (ref=no)	<0.01	0.40
Number of comorbidities at LTCF entry (ref=0-2)	<0.01	<0.01
Marital status (ref=married or de facto)	<0.01	<0.01
Recent BZD or zopiclone use (ref=no)	<0.01	<0.01
Recent antipsychotic use (ref=no)	<0.01	<0.01
Female sex (ref=male sex)	<0.01	<0.01
English not primary language (ref=English primary language)	0.25	0.95
Born outside of Australia (ref=born in Australia)	<0.01	0.22
**Facility factors**		
Remoteness and ownership of LTCF (ref=major city, private provider)	n/a	<0.01
LTCF in SA (ref=VIC)	n/a	0.21

LTCF, long-term care facility, BZD, benzodiazepine, SA, South Australia, VIC, Victoria.
